# A study to design minimum data set of COVID-19 registry system

**DOI:** 10.1186/s12879-021-06507-8

**Published:** 2021-08-09

**Authors:** Javad Zarei, Mohammad Badavi, Majid Karandish, Maryam Haddadzadeh Shoushtari, Maryam Dastoorpoor, Farid Yousefi, Hanieh Raji, Maria Cheraghi

**Affiliations:** 1 Department of Health Information Technology, School of Allied Medical Sciences, Ahvaz Jundishapur University of Medical Sciences, Ahvaz, Iran; 2 Department of Physiology, School of Medicine, Physiology Research Center, Ahvaz Jundishapur University of Medical Sciences, Ahvaz, Iran; 3 Nutrition and Metabolic Diseases Research Center, Ahvaz Jundishapur University of Medical Sciences, Ahvaz, Iran; 4 Air Pollution and Respiratory Diseases Research Center, Ahvaz Jundishapur University of Medical Sciences, Ahvaz, Iran; 5 Department of Biostatistics and Epidemiology, Social Determinants of Health Research Center, Ahvaz Jundishapur University of Medical Sciences, Ahvaz, Iran; 6 Infectious and Tropical Diseases Research Center, Health Research Institute, Ahvaz Jundishapur University of Medical Sciences, Ahvaz, Iran; 7 Department of Infectious Diseases, School of Medicine, Razi Hospital, Ahvaz Jundishapur University of Medical Sciences, Ahvaz, Iran; 8 Social Determinant of Health Research Center, Department of Public Health, School of Health, Ahvaz Jundishapur University of Medical Sciences, Ahvaz, Iran

**Keywords:** Minimum data set, Registry system, COVID-19

## Abstract

**Background:**

From the beginning of the COVID-19 pandemic, the development of infrastructures to record, collect and report COVID-19‏ ‏data has become a fundamental necessity in the world. The disease registry system can help build an infrastructure to collect data systematically. The study aimed to design a minimum data set for the COVID-19 registry system.

**Methods:**

A qualitative study to design an MDS for the COVID-19 registry system was performed in five phases at Ahvaz University of Medical Sciences in Khuzestan Province in southwestern Iran, 2020–2021. In the first phase, assessing the information requirements was performed for the COVID-19 registry system. Data elements were identified in the second phase. In the third phase, the MDS was selected, and in the four phases, the COVID-19 registry system was implemented as a pilot study to test the MDS. Finally, based on the experiences gained from the COVID-19 registry system implementation, the MDS were evaluated, and corrections were made.

**Results:**

MDS of the COVID-19 registry system contains eight top groups including administrative (34 data elements), disease exposure (61 data elements), medical history and physical examination (138 data elements), findings of clinical diagnostic tests (101 data elements), disease progress and outcome of treatment (55 data elements), medical diagnosis and cause of death (12 data elements), follow-up (14 data elements), and COVID-19 vaccination (19 data elements) data, respectively.

**Conclusion:**

Creating a standard and comprehensive MDS can help to design any national data dictionary for COVID-19 and improve the quality of COVID-19 data.

## Background

Although nearly 16 months have passed since the COVID-19 outbreak, and the vaccination is underway in most countries [1], there is still a long way to go to control the pandemic fully [2].

Inequality in vaccine access, insufficient access to vaccines in most poor countries [3–5], and COVID-19 virus mutations are some of the biggest challenges to control the COVID-19 pandemic, especially in developing countries [6]. Besides, there are still many questions about the long-term side effects of recovered patients and the impact of immunity from vaccination [7].

From the beginning of the pandemic, the rapid and surprising outbreak of COVID-19, its complex and unknown nature, has created data collection infrastructures for it, has become a fundamental necessity worldwide [8]. The fundamental step in designing a system to record, collect and report data on a particular disease is determining the target data elements [9]. One of the most well-known tools to determine data elements for disease registration and reporting systems is the minimum data set (MDS) [10]. MDS is a standardized set of data elements defined in a health information system to index the minimum amount of essential data for different users in healthcare sectors [11]. By precisely defining data elements, MDS creates a common language among all those involved in recording, collecting, analyzing, reporting, and interpreting data and ensures the collection of essential data [12, 13]. Furthermore, MDS helps improve medical records documentation, data comparability, data dictionary design, electronic data exchange among different healthcare systems and ultimately improve data quality [12, 14].

This feature has made MDS determination in disease registry systems one of the main steps to implement the system. Based on nature and application, the MDS data elements of a disease can be divided into two general categories of administrative and clinical data. The administrative data usually include demographic, socio-economic, address, phone number, patient referral data, and the main characteristic of a healthcare provider or healthcare center [15–18].

The clinical data vary depending on the type of diseases but generally include diagnosis, past medical history, laboratory findings, medical imaging findings, health interventions, disease course and progress, and outcome [16, 19]. In the case of COVID-19, the data structure is almost the same. Most case report forms, datasets, information systems, and MDSs designed for COVID-19 include patient demographic data, patient’s addresses, and telephone, Profile of healthcare center, how did infected with COVID-19, early signs and symptoms, previous health condition and underlying diseases, COVID-19 testing, CT scan findings, and disease outcome [13, 17, 20–22].

In Iran, at the beginning of the COVID-19 epidemic, the Ministry of Health, as the custodian of the health system, set up two information systems for reporting COVID-19 in healthcare centers. Although these two systems collect basic information about patients with COVID-19, there are problems with data quality and data adequacy to support COVID-19 research [2]. Perhaps this is why several universities of medical sciences in Iran have launched a COVID-19 registry system [22–26].

According to the important role of MDS designing in the success of a disease registry system, this study was performed to design a minimum data set for the COVID-19 registry system in Khuzestan province, southwestern Iran.

## Methods

A qualitative study to design MDS for the COVID-19 registry system was conducted five phases at Ahvaz University of Medical Sciences in Khuzestan Province in southwestern Iran from March 2020 to March 2021.

### Phase 1: Study design and information needs assessment

The purpose of this phase was to answer the following critical questions:

- What is the information that users such as researchers and policymakers of the registry program need for the future?
- According to those requirements, what information of COVID-19 should be recorded?
- What details are available about specific information that is recorded?
- What resources can identify the information?
- What are the minimum data elements?
- Given the urgency of setting up the COVID-19 registry system, what methods can be used to determine MDS?

According to the above-mentioned, a project team was formed under the supervision of the Diseases and Health Outcomes Registry Systems Office in Deputy of Research and Technology of Ahvaz Jundishapur University of Medical Sciences (AJUMS).

The project team reviews the titles of the COVID-19 research proposals submitted to AJUMS as well as interviews with some researchers and experts in the field of disease registry. Moreover, the project team interview with senior managers of Deputy of Research and Technology in AJUMS. In addition, a survey was done on the structure and contents of health information systems such as SIB (an abbreviation for the Persian equivalent of “Integrated Health System”) and hospital information system (HIS), Medical Care Monitoring Center ‏system (a system for reporting COVID-19 cases in hospitals) and the Management of Communicative Disease Prevention and Control system (a system for reporting COVID-19 cases from health centers). Also, the protocol and MDS of previous programs of disease registry at the university were studied. Furthermore, a list of the required information of the COVID-19 registry system were prepared.

In total, the following seven general areas were identified as information needed for the COVID-19 registry system in Khuzestan province:

1. Epidemiological study of COVID-19 prevalence and incidence.
2. The pattern of COVID-19 outbreaks and the factors affecting it.
3. The role of lifestyle in COVID-19 risk.
4. Symptoms, signs, and abnormal medical imaging and laboratory findings of COVID-19.
5. The course of the disease, drug treatment, and the patient’s response to care.
6. Outcomes of COVID-19‏ ‏and the effect of underlying health conditions on its outcomes.
7. Follow up of treated patients.

### Phase 2: Identification of data elements

In the second phase, the goal was to identify the data elements for each of the information needs specified in the previous phase. To identify the data elements, the project team first compiled a list of resources that could contribute to this goal. The resources included articles related to COVID-19, COVID-19 data collection tools, health information systems in Iran, medical records of patients with COVID-19, and expert opinions (such as specialist physicians, health information management specialists, and …). Table 1 describes the information resources used, the search strategy, and the data acquisition method.

**Table 1:** The information sources used for data gathering

Information resources	Data collection methods	Details
Articles	Literature review	In PubMed database and Google search engine the terms “COVID-19” and “SARS-CoV-2” were searched to retrieve related articles The inclusion criteria to select articles were English language, article’s relevance to the objectives, and the possibility of accessing the full text After searching and downloading the article, at the first the title and the abstract were reviewed to choose the relevant articles. At the second stage the full text of selected articles were considered
Data collection tools (forms, data sets, …)	Internet searching	The COVID-19 questioners and reporting forms were googled through the “COVID-19”, “SARS-COV-2”, “Coronavirus disease” terms and in combination with the “Data collection form”, “Data gathering form”, “Minimum data set”, “Data set”, “Data dictionary”, “Questionnaire”, “Form” terms in Persian and English languages The search period was until April 15, 2020
	Request information from experts	A preliminary study showed that some Iranian universities of medical sciences were using a specific paper-based form to register COVID-19 information. Most of the forms were used locally and not available through the internet. However, since the most of Iranian health information management experts who were using the forms are member of social networks such as WhatsApp and Telegram, the project team could access to the some of them
National health information systems	On-site review of information systems	The content and structure of the following Iranian health information systems were reviewed Management of Communicative Disease Prevention and Control system, MCMC system, SIB, and HIS
Patient medical record	Document review	The structure of medical records in Iranian hospitals is hybrid. Part of the patient’s data is documented on paper, and the other part is stored in information systems such as HIS electronically We investigated the data elements of paper-based medical record forms. Furthermore, the process of medical record documentation was reviewed
Experts (medical specialists)	Interview	The attitudes of some medical specialists (pulmonologist, infectious disease, radiologist, and internal medicine) on the minimum data set of COVID-19 have been obtained using the interview

The collections form was used as an instrument. The form contained five sections: the name of the data element, class, application, value, and additional descriptions. The form was designed based on a literature review on MDSs [15, 27–33].

After collecting data, a list of data elements was prepared using the content analysis method. Duplicate or overlap items were removed. After that, similar items were categorized into the main class, subclass, data element, and values. In general, 475 data elements were identified in 18 subclasses and eight main classes.

### Phase 3: Select MDS

At this phase, the purpose was to select MDS from the data elements identified in the previous phase. The expert panel method was used to select MDS. Firstly, a list of medical specialists who are members of the university hospitals of Ahvaz and involved in the care of patients with COVID-19 was prepared. Next, the project team was selected some specialists from the list based on indicators such as interest to join the registry and have a position in the relevant specialized department. The expert panels included medical specialists and some members of the project team. Table 2 shows the demographic characteristics of participants in the expert panels. In the panels, a list of data elements that scaled with the Likert score (1–5) was distributed among participants. During the meetings, data element and their values and feasibility for collection it such as accessibility, method, and time of gathering was discussed. Finally, the primary data elements for MDS were selected.

**Table 2:** Demographic characteristics of participants in the expert panel

Demographic characteristics	Frequency	Present (%)
Sex		
Male	5	50
Female	5	50
Education		
Ph.D.	3	30
Medical specialist	3	30
Fellowship	4	40
Specialty		
Pulmonology	3	30
Infectious disease	1	10
Internal medicine	1	10
Cardiology	1	10
Health information management	1	10
Epidemiology	1	10
Laboratory science	1	10
Emergency medicine	1	10

Furthermore, some new data elements suggested by experts were considered. All data elements with more than 50% of agreement among panelists were elected to apply in the primary MDS.

### Phase 4: Field-testing

Based on primary MDS, we developed web-based COVID-19 registry software (available at http://covid.ajums.ac.ir) using the Rapid Application Development (RAD) technique. In addition to registry software, paper-based forms were designed to help collect data from the hospitals. In the paper forms and registry software, data elements were divided into six top groups: administrative, disease exposure, medical history and physical examination, clinical diagnostic test findings, disease progress and outcome of treatment, and follow-up‏ ‏data. A WhatsApp group that had the project team and experts members was created. The designed forms were shared in the group, and the members were asked to comment on them. After reviewing the members’ comments, the approved comments were applied to the forms.

In the next step, to evaluate the following items, the registry program was tested as a pilot in Razi hospital in Ahvaz city (the capital of Khuzestan province). The evaluated items included structure and content of the designed forms, data values, mandatory fields in the registry software, and identify new data needs. Razi is a 200-bed university hospital considered the main hospital for admitting patients with COVID-19 since late February 2020, when the first positive COVID-19 case was diagnosed in Khuzestan province.

An official letter was sent to Razi Hospital and to make an announcement to invite those interested in participating in the COVID-19 registry program. The Eleven staff of the hospital were selected to participate in the registry system. After training the participants, the registry system was implemented as a pilot for 3 weeks. The participants joined a WhatsApp group to share their opinions and suggestions regarding the data elements, the data collection forms, and the registry software. In addition, two persons of participants were selected to be responsible for monitoring the data collection process and data quality control. They documented all weaknesses and problems related to the data collection process and submitted their recommendations daily. At the end of the pilot, the comments were summarized and submitted to the project manager. Based on the results of the pilot study, some changes were made to MDS, forms, and registry software. The changes included deleting and adding some data elements, renaming the subclass title or data element field name, and modifying their values.

### Phase 5: Evaluate and modify MDS

At the end of May 2020, the COVID-19 registry program was implemented. Six months later, the MDS was evaluated based on the feedback obtained from the initial data analysis and the experiences gotten of the implementation process.

We modified the MDS, removed useless data elements, and added new data elements to the data collection forms and registry software. Finally, when the COVID-19 vaccination was launched in March 2021 in Ahvaz, a new top group was created for recording the vaccination data.

## Results

In the second phase of the study, 375 data elements were categorized into 14 subclasses and eight main classes based on a review of information resources. Then, in the third phase, according to the output of Expert panels, 11 data elements were eliminated, and 25 new data elements were added to the MDS.

Also, according to expert comments, some changes were made in the subclasses, and the number of subclasses increased to 27. For example, the clinical laboratory tests class was divided into five subclasses: hematology test, biochemistry tests, arterial blood gas test, hemostasis test, and other diagnostic tests finding.

After the pilot study, 12 data elements were deleted due to the impossibility of collecting in the routine process of patients with COVID-19 care. Also, 25 new data elements suggested by the participants and approved by the project team were added to the MDS. For example, the ICD10 code and the causes of death recorded in the death certificate were added.

Since to avoid users’ misconceptions and to become data elements transparent, the titles of 13 data elements were renamed.

The values of 26 data elements were also modified in the software. For example, values related to education, job title, and discharge status were changed.

Also, pilot study results showed that the data collection process should be in accordance with the patient care process. Furthermore, to facilitate data collection, the structure of data collection forms and the registry software should be as similar as possible to the medical record forms and HIS.

In the fifth phase, the database was evaluated and modified again. The modifications covered adding a new top group, two main classes, 12 subclasses, and 59 new data elements, as well as removing five subclass and 26 useless data elements. For example, due to the COVID-19 outbreak in Khuzestan province, some data element such as “disease exposure data top group”, and two subclass including “observance of safety measures” and “contact with health care facility” have lost their importance, and finally were removed.

The final MDS for the COVID-19 registry system was divided into eight top groups, 18 main classes, 70 subclasses, and 434 data elements (Table 3). The details of the final MDS are shown in Tables 4, 5, 6, 7, 8, 9, 10 and 11.

**Table 3:** Overall view MDS for the COVID-19 registry system

Rank	Top groups	The number of main classes	The number of sub class	The number of data element
1	Administrative data	2	5	34
2	Disease exposure data	2	7	61
3	Medical history and physical examination data	3	27	138
4	Clinical diagnostic test findings data	3	12	101
5	Disease progress and outcome of treatment data	3	7	55
6	Medical diagnosis and cause of death data	3	6	12
7	Follow-up‏ ‏data	1	4	14
8	COVID-19 vaccination data	1	2	19
Total		18	70	434

**Table 4:** Administrative data about a probable or confirmed cases of COVID-19

Main class	Sub class	Data elements
Individual profile	Demographic	National identity number, Medical record number Patient name, patient surname, father’s name, sex Date of birth, blood groups (types of blood)
	Socio-economic	Marital status, number of family members, Patient ethnicity, educational degree, labor force status, job title, work address (country, province, city, town/village, …)
	Adders	Living status, inhabitancy in residential institution (old people’s home, prison, military camp,…), type of residence (urban, suburban, rural), residential address (country, province, city, town/village, region/neighborhood, …), temporary address (country, province, city, town/village, region/neighborhood, …), zip code, telephone number, mobile phone number, emergency call number
Healthcare Provider Profile	Healthcare facility profile	ID, Healthcare center name, Healthcare center type, Health care center affiliation
	Registrar personnel profile	ID, name, surname, role, specialty

**Table 5:** Disease exposure data about a probable or confirmed case of COVID-19

Main class	Sub class	Data element
Exposure data	Direct contact with an infected person	Has the person had contact with a probable or confirmed case in the 14 days before the onset of symptoms? If yes, The place of encounter: Health care center, hypermarket/shop, gas station, bus/metro, airport/railway station, restaurant/coffee, public administrative area, bank, home, residential institution, weddings, family parties, mourning ceremonies, workplace, other(s), please specify If yes, and the patient knows the person (probable or confirmed case), please state the person’s details: Name and surname, phone number, address
	Travel	Has the case travelled in the 14 days before the symptom onset? If yes: Country, province/state, city, date of departure from the place, details (please explain)
Case finding	Case finding process	Refer to a health care center with symptoms of COVID-19, random screening and identification of the patient, transfer from other health care centers, case finding by tracking who had close contact with previously identified patients, other(s), please specify
	First symptom of disease	Fever, chills, dry cough, nasal congestion, runny nose, sore throat, mucus or phlegm, shortness of breath, constant pain or pressure in chest, tiredness, muscle aches, headache, loss of smell or taste, fatigue, diarrhea, nausea or vomiting, loss of appetite, dry mouth, other(s), please specify, date of first symptom
	First visit to a healthcare center	Type of admission (ambulatory, impatient), date of admission, name of healthcare center
	COVID-19 testing	Type of tests (RT-PCR, antigen/rapid test), sampling method (nasopharyngeal swab, throat swab), date of sampling Test result (positive, negative), laboratory name, date of confirmed disease
	Infection with mutated strains of COVID-19	Infection with mutated strains of COVID-19, type of variant (B.1.1.7, B.1.351, P.1, B.1.427, B.1.429, B.1.617, …)

**Table 6:** Medical history and physical examination data about a probable or confirmed case of COVID-19

Main class	Sub class	Data element
Signs and symptoms at the time of admission	General signs and symptoms	Fever, chills, tiredness, muscle aches, headache, loss of smell or taste, eye congestion, dry mouth, other(s), please specify
	Respiratory signs and symptoms	Dry cough, sneezing, runny nose, dyspnea/tachypnea, constant pain or pressure in chest, nasal congestion, exudative pharyngitis, mucus or phlegm, hemoptysis, other(s), please specify
	Digestive signs and symptoms	Diarrhea, nausea or vomiting, loss of appetite, abdominal pain, constipation, other(s), please specify
	Nervous signs and symptoms	Fatigue, decreased consciousness, Glasgow coma scale/score (GCS), functional limb weakness, other(s), please specify
	Cardiovascular signs and symptoms	Orthopnea, ischemia, angina‏‏, arrhythmias, other(s), please specify
	Others signs and symptoms	Rash on skin, discoloration of fingers or toes, other(s), please specify
Review of systems at the time of admission	Vital signs	Pulse rate, respiratory rate, blood pressure, temperature, level of consciousness
	Thorax	Heart murmurs, wheezing, stridor, pleural friction rub, ventricular gallop, atrial gallop, other(s), please specify
	Abdomen	Tenderness, organomegaly, other(s), please specify
	Limbs	Edema, weak pulse, other(s), please specify
	Height and weight	Height, weight, BMI > 30
	Other	Others signs and symptoms, please specify, additional comment
Underlying conditions and comorbidity	Cardiovascular diseases	Hypertension, heart failure, arrhythmia, ischemic heart disease, other(s), please specify
	Endocrine and metabolic diseases	Type 1 diabetes mellitus, Type 2 diabetes mellitus, unspecified diabetes mellitus If yes, Had the patient’s diabetes be controlled? Metabolic syndrome, other(s), please specify
	Malignant neoplasms	Morphology, primary site, secondary site If yes, Does the patient receive chemotherapy? If yes, Does the patient receive radiotherapy? Additional comment
	Respiratory diseases	Chronic obstructive pulmonary disease (COPD), bronchiectasis, interstitial lung disease (ILD), asthma, other(s), please specify
	Immunodeficiency	HIV/AIDS, congenital immunodeficiency disorders, other(s), please specify
		The patient is being treated with corticosteroids or immunosuppressant medications If yes, Name of medications used, medications dosage
	Chronic neurological or neuromuscular diseases	Cerebral palsy, paraplegia, hemiplegia, multiple sclerosis (MS), old cerebrovascular accident (CVA),other(s), please specify
	Renal diseases	Chronic kidney disease, end stage renal disease (ESRD), other(s), please specify
	Liver diseases	Liver failure, liver cirrhosis, hepatitis (A, B, C), other(s), please specify
	Other important underlying conditions and comorbidity,	Prematurity (infant), mental retardation, other important underlying conditions and comorbidity, please specify
	Pregnancy, childbirth and the puerperium	Pregnancy, gestational age
		Childbirth in the last 6 weeks
	Menopause	Menopause, menopause age
	Smoking, drugs abuse and alcohol	Cigarettes, hookahs, alcohol, drug’s abuse, additional comment
	Medications history	Name of drug, drug dosage, additional comment
Previous history of COVID-19	History of COVID-19	Had a previous history of COVID-19, illness onset date, disease severity status (mild, moderate and severe), additional comment
	History of COVID-19 vaccination	History of COVID-19 vaccination, number of vaccine doses received, date of administration

**Table 7:** Clinical diagnostic test findings data about a probable or confirmed case of COVID-19

Main class	Sub class	Data element
Medical imaging	Chest x-ray‏ ‏findings	Date, normal, ground-glass opacity (GGO), nodular opacities, linear opacities, nodular and linear opacities, cavitation, pneumothorax, pleural effusion, adenopathy, atelectasis, patchy, cardiomegaly, consolidation, other(s), please specify
	CT scan‏ ‏findings	Date, normal, ground-glass opacity‏ ‏(GGO), tree in bud: (branching nodular and linear opacities), crazy-paving, cavitation, pneumothorax, pneumothorax, pleural effusion, adenopathy, atelectasis, patchy, consolidation, cardiomegaly
		Pulmonary involvement detail: Right lung only,‏ ‏left lung only, ‏both lung, ‏upper zone, middle zone, lower zone
		Diagnosis, additional comment
Cardiology diagnostic tests and procedures	Electrocardiogram (ECG or EKG) findings	Date, normal, sinus bradycardia, sinus tachycardia, arrhythmias, long QT, other(s), please specify
	Echocardiography findings	Date, normal, congestive heart failure (CHF), coronary artery disease (CAD), elevated pulmonary pressure (PAP), mitral regurgitation [34], pulmonary regurgitation (PR), tricuspid regurgitation (TR), other(s), please specify
Clinical laboratory tests	Hematology test findings	Date, hematocrit (HCT), hemoglobin (Hgb, Hb), mean corpuscular volume (MCV), mean corpuscular hemoglobin concentration (MCHC), red cell distribution width (RDW) White blood cell (WBC) count, lymphocytes, neutrophils, eosinophils, monocytes, basophils
	Biochemistry tests findings	Date, creatine, serum glutamic oxaloacetic transaminase (SGOT/AST), serum glutamic pyruvic transaminase (SGPT/ALT), alkaline phosphatase, bilirubin, blood urea nitrogen (BUN), N-terminal pro brain natriuretic peptide (NT-proBNP), procalcitonin (PCT), high-density lipoprotein cholesterol (HDL-C), troponin, albumin, lactate
	Arterial blood gas test findings	Date, PaO_2_, PaCO_2_, pH, HCO_3_, O_2_CT, O_2_Sat, saturation
	Hemostasis test	Date, prothrombin time (PT), partial thromboplastin time (PTT), mean platelet volume (MPV), d-dimer, International normalized ratio (INR)
	Other diagnostic test	Date, C-reactive protein (quantitative), interleukin 6 (IL-6), other(s) diagnostic test, please specify

**Table 8:** Disease progress and outcome of treatment data in a probable or confirmed case of COVID-19

Main class	Sub class	Data element
Admission to hospital	Hospitalization	Need for hospitalization, first date of admission to hospital, discharge date, discharge status, hospital name, hospital type, hospital ID/code
	Care in an intensive care unit	Receive care in an intensive care unit, Length of stay (LOS) in ICU
	Receive ventilation and intubation	Receive mechanical ventilation, the number of ventilator days, need to intubation, the number of intubation days
OVID-19 Medications	The main medications prescribed	Date, chloroquine phosphate, hydroxychloroquine, kaletra (lopinavir/ritonavir), ribavirin, remdesivir, avipiravir, oseltamivir (tamiflu), Atazanavir/ritonavir, plasma transfusion, other(s), please specify, additional comment
	The auxiliary medications prescribed	Date, naproxen, azithromycin, IVIG, sofosbuvir/daclatasvir (Sovodak), other(s), please specify, additional comment
Disease complications and outcome	Systemic complications	Sepsis/septic shock, multisystem inflammatory syndrome in children (MIS-C), multiorgan failure, cytokine release syndrome, acute kidney injury (AKI), acute liver injury, hepatitis
	Respiratory complications	Pneumonia, ‏acute respiratory distress syndrome (ARDS), respiratory failure
	Cardiovascular complications	Myocardial infarction, heart failure, arrhythmias, acute coronary syndrome, endothelialitis, vasculitis, viral myocarditis, other cardiovascular complications
	Hematologic complications	Disseminated intravascular coagulation (DIC), thrombocytopenia, leukocytosis, leukocytopenia, pulmonary embolism (PE), deep vein thrombosis (DVT)
	Neurologic complications	Cerebrovascular accident (CVA), meningitis, encephalopathy, other neurologic complications
	Other complications	Date, the name of complications, additional comment
	Outcome	Recovered/healthy, death, not recovered, other(s), please specify

**Table 9:** Medical diagnosis and cause of death data in a probable or confirmed case of COVID-19

Main class	Sub class	Data element
Medical diagnosis	Primary diagnosis/impression	Date, Primary diagnosis ICD10 code
	final diagnosis	Principal diagnosis ICD10 code, secondary diagnoses ICD10 code
Medical procedure	Medical procedure	Principal procedure code, other procedure code
Cause of death	Direct cause of death	Medical diagnosis, ICD10 code
	Intermediate causes of death	Medical diagnosis, ICD10 code
	Underlying cause of death	Medical diagnosis, ICD10 code

**Table 10:** Follow-up data in a probable or confirmed case of COVID-19

Main class	Sub class	Data element
Patient’s follow-up	Patient status until 2 weeks after discharge/quarantine	*Quarantine location* (home, recovery camp and others) *Patient status in the 1st week* (isolation/quarantine according to the standards for COVID-19 patients, quarantine without safety protocols, return to work, exacerbation of the disease, unspecified) *Patient status in the 2nd week* (isolation/quarantine according to the standards for COVID-19 patients, quarantine without safety protocols, return to work, exacerbation of the disease, unspecified)
	Symptoms of the disease after discharge	Dry cough, shortness of breath, constant pain or pressure in chest, tiredness, loss of smell or taste, loss of appetite, other(s), please specify, date of the last symptom
	Contact details	Date of contact, delivery mode, responsive person (if the respondent is not patient himself/herself), reporting person

**Table 11:** COVID-19 vaccination‏ ‏data

Main class	Sub class	Data element
COVID-19 vaccination‏ ‏data	COVID-19 Vaccine Administration data	COVID-19 vaccine (Oxford-AstraZeneca, Sputnik V, Sinopharm, etc.), date the first dose administration, date the second dose administration, places to provide the COVID-19 vaccine (health center ID, name, address)
	Vaccine side effect	Date, pain at the injection site, tiredness, headache, muscle or joint pain, chills, fever, fatigue, nausea and vomiting, diarrhea, swollen lymph nodes, severe allergic reaction (difficulty breathing, dizziness, swelling of the face and throat, …), thrombosis, thrombocytopenia, other(s), please specify

In general, the administrative data group includes two main classes, five subclasses, and 34 data elements (Table 4).

In general, the disease exposure data group includes two main classes, seven subclasses, and 61 data elements (Table 5).

In general, the medical history and physical examination data group embraces four main classes, 27 subclasses, and 138 data elements (Table 6).

In general, the clinical diagnostic test findings data group consists of three main classes, 12 subclasses, and 101 data elements (Table 7).

In general, the disease progress and outcome of treatment data group includes three main classes, 12 subclasses, and 55 data elements (Table 8).

In general, medical diagnosis and cause of death data group consists of three main classes, six subclasses, and 12 data elements (Table 9).

In general, follow-up‏ ‏data group embraces one main class, three subclasses, and 14 data elements (Table 10). 

In general, the COVID-19 vaccination‏ ‏data group includes one main class, two subclasses and, and 19 data elements (Table 11).

## Discussion

A study on the coronavirus family showed that creating a data set can help disease control intervention [35]. The lack of MDS is one of the technical barriers to data sharing in public health [36]. Therefore, from the beginning of the COVID-19 outbreak, World Health Organization has recommended a “Revised case reporting form for 2019 Novel‏ ‏Coronavirus of confirmed and probable cases” to report COVID-19 cases and has requested national authorities to design MDS for reporting COVID-19 based on the form [37]. However, that form may not be suitable for the COVID-19 registry system. Since the many aspects of COVID-19 were unknown at the beginning of the pandemic, there were a lot of questions about the disease in the minds of scientists and health policymakers. Therefore, the COVID-19 registry system should provide comprehensive information [2]. This study tried to fulfill that requirement. The approach used to design MDS included identifying the required data elements, the initial design of MDS, the pilot test, and modification of MDS based on the results obtained from the implementation. Stanfill et al., in the study entitled “Health information management best practices for quality health data during COVID-19 global pandemic”, had the same approach as our study. They emphasized identifying the data elements, considered the expected results, and evaluated the output data to justify data elements [38].

In the MDS for the COVID-19 registry system, 396 data elements were identified in eight top groups, 17 main classes, and 67 subclasses. The first top group included administrative data that are commonly used to identify and register patients, identify a healthcare institution, reimburse the costs of healthcare services [39, 40], do medical research, and carry out a survey on the outcome and quality of healthcare [21, 41].

The second top group was disease exposure data. These data can help to determine the method of individual transmission of disease and identify early signs and symptoms. Therefore, in order to show how the patient was exposed to infection, the exposure data was used in the coronavirus family data set. For instance, COVID-19 forms and dataset [37], British government MDS form for MERS disease [42], and MDS for SARS [35].

Another main issue with COVID-19 is the new variants of the virus. One of the principal concerns about the coronavirus variants is whether the mutations could affect treatment and prevention or not [43]. The importance of recording COVID-19 variants led to consider a subclass for infection with mutated strains of COVID-19 in the fifth phase of the study.

The third top group of MDS was dedicated to medical history and physical examination data. It included 22 subclasses for recording signs and symptoms of COVID-19 at the time of admission, as well as documenting the patient’s underlying medical conditions. From the beginning of the COVID-19 outbreak, much attention was paid to identifying the signs and symptoms of the disease and distinguishing it from other infectious diseases. Moreover, since the early studies in China showed that underlying medical conditions impact on COVID-19 outcome [44], in all forms, MDS or dataset, and health information systems related to COVID-19, the data elements of underlying medical conditions were considered. Furthermore, the information needs assessment phase indicated that most titles of COVID-19 research proposals submitted to AJUMS focused on the effect of underlying medical conditions on the outcome of COVID-19. In addition, most of the data elements in the two national COVID-19 recording and reporting systems of the Iranian Ministry of Health (Management of Communicative Disease Prevention and Control and MCMC system) are dedicated to recording the sign and symptoms and underlying diseases. Therefore, in the MDS of the COVID-19 registry system, a classified approach was used to define the symptoms and underlying diseases to record them more accurately.

The fourth top group of MDS was related to clinical diagnostic test findings. The purpose of the three main classes in this group was to record abnormal medical imaging, laboratory, and cardiology diagnostic tests in patients with COVID-19, especially those hospitalized. One of the most important of the data was medical imaging data. The findings of CT scan and chest x-ray play an essential role in the diagnosis, the clinical course of the disease, and the choice of treatment for patients with COVID-19 [45–47].

The importance of imaging data has encouraged the Iranian Ministry of Health, in collaboration with the Iranian Society of Radiology to propose a standard form for reporting medical imaging data in patients with COVID-19 [48]. In the same manner, the Radiological Society of North America (RSNA) also has provided a standard classification for reporting CT scan findings on COVID-19 [49]. The study indicated that laboratory diagnostic data and cardiology diagnostic tests were other essential data that medical specialists emphasized on registering them. The reason for this attention may be the importance of the data elements in the treatment of inpatients with COVID-19 [50–54].

Disease progress and outcome of treatment data were the next top group of MDS. The data shows a period of the disease and the outcome of care. If the data combine with other data such as age, sex, underlying medical conditions and comorbidity, and clinical diagnostic test findings, they can facilitate many statistical analysis and artificial intelligence data modeling that can be applied in reliable prediction about the outcome of the disease based on extracting hidden relationships among the clinical and non-clinical data [55–57].

Therefore, 52 data elements were considered to record clinical course (such as hospitalization), disease complications, treatment measures, and disease outcome. The disease complications and outcomes were considered essential data in other forms, MDSs, and data sets related to COVID-19. For example, Shanbehzadeh and Kazemi-Arpanahi, in their study entitled “Development of minimal basic data set to report COVID-19”, proposed two subclasses for data elements of disease complications and outcomes [13].

Another questionable issue related to the COVID-19 is the effectiveness of various medications on the treatment outcome. Although the forms and databases for recording and reporting COVID-19 do not usually mention the medications used to treat the patient, we have considered a separate class for medications because the topic is important for medical research.

The data elements related to medications may help to identify the effects of different medications in the treatment of COVID-19.

The top group for medical diagnosis and cause of death data was considered to record the relationship between primary and final diagnosis and the cause of death in patients with COVID-19.

The group included diagnostic (based on WHO’s ICD-10 classification system), causes of death, and medical procedures codes (based on ICD9CM Volume 3).

Clinical coding of medical records can help to manage statistical data about diseases and causes of death at the national or international levels [58]. Therefore, that data could facilitate retrieving information about patients with COVID-19 in the future. Most studies conducted to design MDS of diseases have suggested using diagnostic data elements [16, 27, 29].

Coinciding with the COVID-19 Pandemic, the WHO proposed new ICD codes for the disease to help classify and report it. WHO also published a guideline for recording and coding the causes of death due to COVID-19 [59]. In addition to WHO efforts, some countries have established a process to record COVID-19 coding data. For example, in the Irish COVID-19 data set, “diagnosis concepts” is considered for recording diagnostic data [60]. Also, in the United States, several guidelines were published by the CDC, AHIMA, and AMA regarding the recording and coding of diagnoses and medical procedures for COVID-19 [13, 61, 62]. As the same manner, Iran’s Ministry of Health has already issued three guidelines for coding COVID-19 [48].

The top group was follow-up data. In the group, data related to patient monitoring up to 2 weeks after discharge from the hospital or quarantine at home was recorded.

The patient follow-up is important for two reasons: firstly, to ensure that the signs and symptoms of the disease improve, and secondly, to track people who are in close contact with the patient. Therefore, the patient follow-up is essential protocols in controlling COVID-19 especially for a patient who is in quarantine at home.

Another fundamental way to effectively control the disease is vaccination. COVID-19 vaccination started in March 2021 in Ahvaz. Therefore, at the beginning of vaccination, we considered a top group of MDS to record its data. Recording vaccination information is valuable in several ways. Firstly, the data can be used to evaluate the effectiveness of different vaccines. Secondly, they help to evaluate the vaccination effectiveness on disease control. Finally, the data can aid in assessing the side effects of vaccines.

## Conclusion

The MDS designed in the present study has tried as much as possible to cover the essential data for the COVID-19 registry system. Using a comprehensive MDS and a systematic approach to data collection of COVID-19 can provide valuable information for health policymakers, researchers, and clinical specialists.

Given the importance of having accurate, complete, and up-to-date information on COVID-19 disease control, it strongly recommends that different countries should design a comprehensive national MDS for COVID-19. The methodology used in this study can also help other developing countries that do not have a comprehensive infrastructure for disease recording and reporting systems to implement similar disease registry systems.

## Limitation of study

At the beginning of the project, there were few related studies to our topic. Also, due to the difficulty in long-term follow-up of patients, data elements have not been considered for long-term COVID-19 complications.

### Further study

We recommend that future studies can focus on the effectiveness of the COVID-19 registry system in data quality. Also, we strongly recommend that an international MDS be designed for the long-term complications of COVID-19.

## Data Availability

The datasets used during the current study are available from the first author on reasonable request.

## Abbreviations

AHIMA: American Health Information Management Association
AJUMS: Ahvaz Jundishapur University of Medical Sciences
AKI: Acute kidney injury
AMA: American Medical Association
ARDS: Acute respiratory distress syndrome
BMI: Body mass index
BUN: Blood urea nitrogen
CAD: Coronary artery disease
CDC: Center for Disease Control
CHF: Congestive heart failure
CKD: Chronic kidney disease
COPD: Chronic obstructive pulmonary disease
CRP: C-reactive protein
CVA: Cerebra vascular accident
DIC: Disseminated intravascular coagulation
GCS: Glasgow coma scale/score
Hb: Hemoglobin
HCT: Hematocrit
HIS: Hospital information system
ICTV: International Committee on Taxonomy of Viruses
ILD: Interstitial lung disease
INR: International normalized ratio
LDH: Lactic acid dehydrogenase
LOS: Length of stay
MCHC: Mean corpuscular hemoglobin concentration
MCMC: Medical Care Monitoring Center System
MCV: Mean corpuscular volume
MDS: Minimum data set
MS: Multiple sclerosis
NT-proBNP: N-terminal pro brain natriuretic peptide
PCT: Procalcitonin
PR: Pulmonary regurgitation
PT: Prothrombin time
PTT: Partial thromboplastin time
RAD: Rapid Application Development
RDW: Red cell distribution width
TR: Tricuspid regurgitation
WBC: White blood cell
WHO: World Health Organization
